# Comparison of Survival and Risk Factors of Differentiated Thyroid Cancer in the Geriatric Population

**DOI:** 10.3389/fonc.2020.00042

**Published:** 2020-02-03

**Authors:** Lujiao Yu, Hong Hong, Jinyu Han, Sean X. Leng, Haiyan Zhang, Xu Yan

**Affiliations:** ^1^Department of Geriatrics, The First Hospital of China Medical University, Shenyang, China; ^2^Division of Geriatric Medicine and Gerontology, Johns Hopkins University School of Medicine, Baltimore, MD, United States; ^3^The VIP Department, School and Hospital of Stomatology, China Medical University, Liaoning Provincial Key Laboratory of Oral Diseases, Shenyang, China

**Keywords:** differentiated thyroid carcinoma, elderly people, risk factor, SEER database, Disease-specific survival

## Abstract

**Purpose:** The incidence rate of differentiated thyroid cancer (DTC), the most common type of thyroid cancer, has increased in the past two decades. The present study analyzed the clinical and pathological characteristics of DTC, and discussed the risk factors for survival in elderly age-risk DTC patients.

**Methods:** Elderly patients who were diagnosed with DTC, and subsequently underwent surgery for DTC, were identified from the SEER database (1988–2008). Based on histology, these patients were divided into C-PTC, FV-PTC, and FTC. The clinical characteristics, pathological features, and treatments undertaken were compared among these patients. Cox proportional hazards analysis was performed to evaluate the risk factors to disease-specific survival (DSS).

**Results:** In elderly DTC patients, FV-PTC shows intermediate tumor features compared to C-PTC and FTC, but presented a better outcome. Being male, African-American, tumors sized bigger than 4 cm, extrathyroidal extension, lymph node metastasis, and distant metastasis, were all strong risk factors for DSS in elderly DTC patients (all *p* < 0.05). No difference was found between lobectomy and total thyroidectomy with respect to DSS, and radiation therapy conferred no apparent advantage with respect to DSS (both *p* > 0.05).

**Discussion:** Patients with FV-PTC needed more specific histology cataloging and risk assessment, suggesting conservative therapy. Risk stratification should be paid attention to, and treatment should be individualized for elderly patients.

## Introduction

Thyroid cancer is one of the most prevalent tumors worldwide. In recent years, new cases of thyroid cancer within the United States have been increasing (approximately 37,200 in 2009 vs. 63,000 in 2014) (1). Differentiated thyroid cancer (DTC), including papillary thyroid cancer (PTC) and follicular thyroid cancer (FTC), account for more than 90% of thyroid cancers (2). Although incidence rates of DTC increased over the past decade, most DTC patients had a good prognosis, especially PTC patients: 5 year survival in PTC is >97% in general (3–6).

The increasing of incidence has been mainly attributed to the growth of PTC cases (7, 8). PTC is not a homogenous group. More than half of PTC tumors are classified as “classical” PTC (C-PTC). The most common and fastest growing subtype of PTC is the follicular variant of papillary thyroid cancer (FV-PTC), which belongs to the “non-classical” subgroup, and accounts for 24–33% of all PTCs (8, 9). FV-PTC refers to thyroid cancer that has the nuclear feature of PTC but the growth behavior of FTC. Thus, FV-PTC is believed to be an intermediate tumor type between PTC and FTC. Some studies have focused on the disease features and outcomes of FV-PTC due to its unique tumor feature and high prevalence. Certain invasive histology subtypes of PTC that lead to a high mortality and have a high recurrence rate, such as the columnar cell variant and the diffuse sclerosing variant, were excluded from this study due to the low incidence rate of such variants. The survival of FTC patients is compromised compared to that of PTC patients, they therefore attract our attention especially.

Tumor size, lymph node metastasis, and distant metastasis are all risk factors for DTC (3). Previous studies have shown that these risk factors have different effects on different histology subtypes (4, 9). Therefore, comparing the clinical-pathological features among the different subtypes of DTC should further elucidate the risk factors and prognosis of DTC, thus helping guide clinical decisions regarding DTC cases.

Age is a vital risk factor for DTC. Most studies on the cancer-specific survival of patients with DTC have determined age>=45 to be a major risk factor (5, 6, 10), and the AJCC stage system (TNM) also sets age 45 as a factor in the tumor stratification stage. With that said, few studies have been conducted on the prognosis of elderly patients with DTC. In fact, most cancer studies have primarily focused on adults (>=18 years old), and very few have specifically targeted the elderly. There are different cut-off values for “elderly”; 65 years-old is the most widely accepted (11, 12). It thus remains unclear whether the types of surgery or other treatments used to treat adults with DTC would be optimal for elderly DTC patients. Moreover, the prognostic factors for elderly patients with DTC remain unknown.

## Methods

### Data Selection

All data was obtained from the National Cancer Institute's Surveillance, Epidemiology, and End Results (SEER) Program database. SEER is a source of information concerning cancer incidence and cancer survival in the United States. The database is comprised of 18 cancer registries derived from Atlanta, Connecticut, Detroit, Greater California, Greater Georgia, Hawaii, Iowa, Kentucky, Los Angeles, Louisiana, New Jersey, New Mexico, Rural Georgia, San Francisco-Oakland, San Jose-Monterey, Seattle-Puget Sound, Utah, and the Alaska Native Tumor Registry, together representing approximately 28% of the total US population (13). From these 18 registries, we utilized the data derived from 1988 to 2008, inclusive (November 2015 submission), which is thought to be representative of the DTC population in the U.S.

Cases of confirmed thyroid cancer were identified using the primary tumor site code of C73.9 (thyroid gland) in combination with the International Classification of Disease for Oncology, 3rd Edition (ICD-O-3 codes) with the following histology codes: C-PTC (8050, 8260, 8341-8343), FV-PTC (8340), FTC (8330-8332, 8335). Age of the subjects used for the study was restricted to 65 years or above. All the diagnoses were confirmed histology-positive. Only cases with active follow-up were selected, and those with autopsy- or death certificate-only were excluded from the study. Patients who had more than one primary malignancy were excluded.

Clinical information collected on the patients included age, sex, race, marital status, survival time, surgical method, and status of radiation. Pathologic variables of interest included tumor size, lymph node metastasis, extra-thyroidal extension, distant metastasis, and SEER stage. Extra-thyroidal extension was defined as tumor invasion beyond the thyroid capsule. Since surgery was recommended to be the first choice in thyroid cancer therapy (1), this study focused on the effect of surgical method on prognosis, instead of on the effect of performing surgery or not.

Surgical methods were divided into total thyroidectomy (including total and subtotal thyroidectomy) and lobectomy (all surgical methods other than the former).

### Statistical Analysis

We summarized the clinical information and tumor features of C-PTC, FV-PTC, and FTC, and compared these data among the three groups. Continuous variables were presented as mean ± standard deviation (SD); categorical variables were presented as frequency (percentage). Within each of the three DTC subgroups, categorical variables were analyzed using the chi-squared test, while continuous variables were analyzed using one-way analysis of variance (ANOVA). Disease-specific survival (DSS) and overall-cause survival (OS) were determined by survival analysis, and survival equality was assessed using the log-rank test. The Cox proportional hazards model was used to identify the risk factors that correlated with DSS. All the analyses were performed using Stata 14.0 (StataCorp, LP, USA). *p* < 0.05 (two-sided) was considered to be statistically significant.

## Results

### Univariate Analyses

There were 7,784 cases which met the criteria for analysis. Among them, 169 cases did not undergo surgery for DTC, and were excluded from the study. With respect to the reasons for not having undergone surgery for DTC, there were 123 cases (72.8%) for which surgery was not recommended, 34 cases (20.1%) with an unknown cause, 9 cases (5.3%) in which the patient refused surgery, and 3 cases (1.8%) in which the patient died prior to surgery.

Among the total of 7,615 cases that were selected, 4,472 cases were C-PTC (58.7%), 2,266 were FV-PTC (29.8%), and 877 were FTC (11.5%). The demographic, clinical, treatment, and tumor characteristics of the three DTC groups are summarized in Table 1. The mean age was similar in C-PTC, FV-PTC, and FTC (72.5 ± 6.1, 72.2 ± 5.8, and 73.6 ± 6.3, respectively). Patients with FTC were slightly older than those with PTC (*p* < 0.001). Female and Caucasians were the most prevalent among the three DTC groups. As for marital status, the difference between C-PTC and FV-PTC was not significant, and more than half of people in each group (57.7, 56.7, and 50.5%) were married and/or had a partner.

**Table 1:** Summarization of clinic-pathological data in patients of C-PTC, FV-PTC, and FTC.

		**C-PTC(4472)**	**FV-PTC(2266)**	**FTC(877)**	
Age		72.5877	72.2877	73.6877	0.000^*^
Gender	Male(%)	1,184 (26.5)	562 (24.8)	297 (33.9)	
	Female(%)	3,288 (73.5)	1,704 (75.2)	580 (66.1)	0.000^*^
Race	White(%)	3,648 (81.6)	1,864 (82.3)	675 (77.0)	
	Black(%)	198 (4.4)	176 (7.8)	102 (11.6)	
	Asian/Pacific islander(%)	613 (13.7)	217 (9.6)	97 (11.1)	
	Unknown(%)	13 (0.3)	9 (0.4)	3 (0.3)	0.000^*^
Marital	Married(%)	2,578 (57.7)	1,285 (56.7)	443 (50.5)	
	Single(%)	1,737 (38.9)	905 (39.9)	407 (46.4)	
	Unknown(%)	157 (3.5)	76 (3.4)	27 (3.1)	0.002^*^
Surgery	Lobectomy(%)	821 (18.4)	470 (20.7)	241 (27.5)	
	Thyroidectomy(%)	3,546 (79.3)	1,760 (77.7)	602 (68.6)	
	Unknown(%)	105 (2.4)	36 (1.6)	34 (3.9)	0.000^*^
Radiation	YES(%)	2,041 (45.6)	1,077 (47.5)	453 (51.7)	
	No(%)	2,381 (53.2)	1,165 (51.4)	416 (47.3)	
	Unknown(%)	50 (1.1)	24 (1.1)	8 (0.9)	0.024^#^
Tumor size	<=1 cm(%)	1,646 (36.8)	794 (35.0)	43 (4.9)	
	1–4 cm(%)	1,938 (43.3)	1,004 (44.3)	386 (44.0)	
	>4 cm(%)	458 (10.2)	290 (12.8)	313 (35.7)	
	Unknown(%)	430 (9.6)	178 (7.9)	135 (15.4)	0.000^*^
Lymph nodes examination	Yes(%)	1,644 (36.8)	634 (28.0)	134 (15.3)	
	No(%)	2,681 (60.0)	1,574 (69.5)	725 (82.7)	
	Unknown(%)	147 (3.3)	58 (2.6)	18 (2.1)	0.000^*^
Lymph nodes positive	Yes(%)	944 (21.1)	268 (11.8)	44 (5.0)	
	No(%)	803 (18.0)	414 (18.3)	100 (11.4)	
	Unknown(%)	2,725 (60.9)	1,584 (69.9)	733 (83.6)	0.000^*^
Lymph nodes metastases	Yes(%)	994 (22.2)	278 (12.3)	59 (6.7)	
	No (%)	3,028 (67.7)	1,770 (78.1)	644 (73.4)	
	Unknown(%)	450 (10.1)	218 (9.6)	174 (19.9)	0.000^*^
Extrathyroidal extension	Yes (%)	1,296 (29.0)	433 (19.1)	231 (26.3)	
	No(%)	3,083 (68.9)	1,807 (79.7)	627 (71.5)	
	Unknown(%)	93 (2.1)	26 (1.2)	19 (2.2)	0.000^*^
Distant metastasis	Yes(%)	159 (3.6)	83 (3.7)	124 (14.1)	
	No(%)	3,873 (86.6)	1,990 (87.8)	639 (72.9)	
	Unknown(%)	440 (9.8)	193 (8.5)	114 (13.0)	0.000^*^
Stage	Localized(%)	2,548 (57.0)	1,516 (66.9)	382 (43.6)	
	Regional(%)	1,607 (35.9)	623 (27.5)	350 (39.9)	
	Distant(%)	258 (5.8)	112 (4.9)	134 (15.3)	
	Unstaged(%)	59 (1.3)	15 (0.7)	11 (1.3)	0.000^*^

* means p < 0.01;

# *means p < 0.05*.

The rate of total thyroidectomy was 79.3%, 77.7%, and 68.6% for C-PTC, FV-PTC, and FTC, respectively. Patients with FTC were more likely to have radiation therapy in combination with surgery in comparison to the other two disease groups (45.6%, 47.5%, and 51.7% for C-PTC, FV-PTC, and FTC, respectively, *p* = 0.024). The method of radiation therapy is not a detail described in SEER data.

With respect to tumor features, the tumor size of FTC patients was significantly larger than that of the other two groups (*p* < 0.001). Moreover, the rate of extra-thyroidal extension (29.0% for C-PTC, 19.1% for FV-PTC, and 26.3% for FTC) and lymph nodes metastasis (22.2% for C-PTC, 12.3% for FV-PTC, and 6.7% for FTC) were highest in C-PTC, while distant metastasis rate (3.6% for C-PTC, 3.7% for FV-PTC, and 14.1% for FTC) was highest in the FTC group. Notably, since the follow-up period was over 10 years, AJCC TNM staging had undergone several revisions. Therefore, we used the SEER stage to assess the prognosis of tumors instead of the TNM stage. We found that patients with FTC were at a more advanced stage than patients with either C-PTC or FV-PTC.

**Figure 1:**
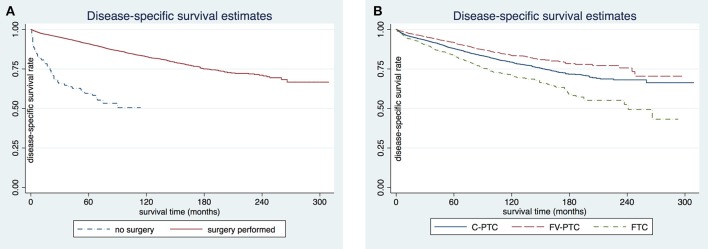
Comparison of disease-specific survival rates in patients older than 65 years by surgery **(A)** or histology subgroup **(B)**.

**Figure 2:**
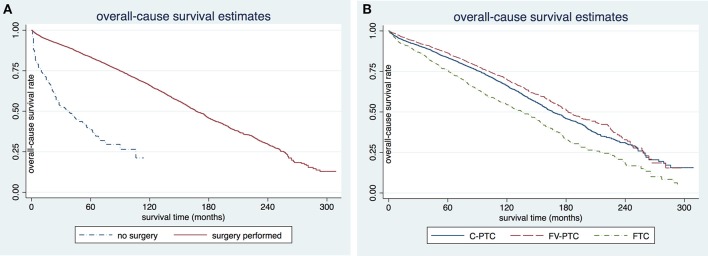
Comparison of overall-cause survival rates in patients older than 65 years by surgery **(A)** or histology subgroup **(B)**. C-PTC, papillary thyroid cancer; FV-PTC, follicular variant of papillary thyroid cancer; FTC, follicular thyroid cancer.

The disease-specific survival (DSS) and overall survival (OS) for each of the three DTC groups are given in Table 2. The mean follow-up time for the entire study cohort was 97.7 ± 0.6 months (96.8 ± 54.0 months in C-PTC, 101.6 ± 52.6 months in FV-PTC, and 92.4 ± 58.5 months in FTC). DSS and OS were presented by status of surgery and histology (Figures 1, 2). The survival rate among the three groups was significantly different, as shown by the log-rank test (*p* < 0.001).

**Table 2:** Comparison of survival rates among patients with DTC.

	**C-PTC** **(*N* = 4472)**	**FV-PTC** **(*N* = 2266)**	**FTC** **(*N* = 877)**	***P***
5 year disease-specific survival	90.7%	92.9%	86.7%	0.000^*^
5 year overall survival	83.4%	86.3%	75.4%	0.000^*^
10 year disease-specific survival	82.9%	85.5%	74.3%	0.000^*^
10 year overall survival	66.1%	69.8%	54.4%	0.000^*^
20 year disease-specific survival	71.2%	77.6%	55.0%	0.000^*^
20 year overall survival	30.8%	32.8%	17.8%	0.000^*^

* *means p < 0.05*.

### Multivariate Analyses

We performed Cox proportional hazards regression analysis for the DTC patients as a whole (Table 3). Then, Cox analysis was performed for each subtype of thyroid cancer to determine the independent predicators of each DTC subtype (Table 4). The regression analysis indicated that gender, race, marital status, histological type, tumor size, lymph node metastasis, extra-thyroidal extension, and distant metastasis were all factors that influenced DSS of DTC patients (*p* < 0.05). In particular, being female, an Asian/Pacific islander, or married, all individually translated to a longer DSS.

**Table 3:** Cox proportional hazards analysis of risk factors among patients with DTC.

	**DSS**		
	**HR**	**95%CI**	***p***
**Gender**			
Female	ref		
Male	1.416	1.243–1.615	0.000^*^
**Race(ref. white)**			
White	ref		
Black	1.301	1.041–1.626	0.020^#^
Other	0.782	0.654–0.936	0.007^*^
**Married status**			
Single	ref		
Married	0.871	0.767–0.990	0.034^#^
**Histology**			
C-PTC	ref		
FTC	1.300	1.089–1.552	0.004^*^
**Size**			
<=4 cm	ref		
>4 cm	1.469	1.204–1.793	0.000^*^
**Nodal metastasis**			
No	ref		
Yes	2.131	1.839–2.469	0.000^*^
**Extension**			
No	ref		
Yes	2.078	1.804–2.394	0.000^*^
**Distant metastasis**			
No	ref		
Yes	2.419	1.974–2.965	0.000^*^

* p < 0.01;

# *p < 0.05*.

*The model includes age, gender, race, marital status, extent of thyroidectomy, radioactive therapy status, tumor size, lymph nodes metastases, extrathyroidal extension, and distant metastasis*.

**Table 4:** Cox proportional hazards analysis of risk factors among patients with C-PTC, FV-PTC, and FTC.

**Risk factor**	**C-PTC HR (95%CI)**	**FV-PTC HR (95%CI)**	**FTC HR (95%CI)**
Male	1.374(1.158–1.631)^*^	1.587(1.210–2.082)^*^	1.350(0.981–1.859)^&^
Race(ref. White)			
Black	1.546(1.085–2.205)^#^	1.384(0.932–2.054)^&^	1.032(0.662–1.607)^&^
Other	0.879(0.707–1.091)^&^	0.646(0.411–1.015)^&^	0.612(0.380–0.985)^#^
Nodal metastasis	2.333(1.941–2.805)^*^	1.581(1.146–2.180)^*^	2.558(1.600–4.088)^*^
Extrathyroidal extension	1.956(1.627–2.352)^*^	2.060(1.527–2.780)^*^	2.700(1.905–3.826)^*^
Distant metastasis	2.607(1.981–3.431)^*^	2.452(1.612–3.729)^*^	1.872(1.151–3.045)^#^
Marital status (ref. Single)			
Married	0.875(0.741–1.033)^&^	0.878(0.678–1.138)^&^	0.850(0.623–1.159)^&^

* p < 0.01;

# p < 0.05;

& *p >= 0.05*.

As for the subgroup Cox regression analysis, the results were different from the above. The male gender was a risk factor only for PTC (*p* < 0.01), and it did not affect the DSS of FTC. Although tumor size was also an important index, it did not show a significant effect on the DSS of any of the three subtypes based on subgroup analysis. Lymph node metastasis, extra-thyroidal extension, and distant metastasis are all strong predicators that increased the disease-specific mortality (DSM) of each of the three DTC groups. Based on subgroup analysis, marital status did not affect the DSS of any of the three subtypes. In both the analysis of DTC and the subgroup analysis of the three subtypes, total thyroidectomy, in comparison to lobectomy, did not show any apparent advantage in terms of lengthening DSS (*p* > 0.05). Likewise, radiation therapy did not affect the DSS of the three subgroups (*p* > 0.05).

## Discussion

PTC and FTC are two major types of well-differentiated thyroid cancer and have excellent prognosis. FV-PTC is the major variant of PTC, and has intermediate clinical-pathological features and prognosis, between those of PTC and those of FTC (14). The incidence rate of FV-PTC has risen rapidly within the past two decades (3, 4, 7–9). In this study, FV-PTC patients accounted for 33.6% of all PTC cases and 29.8% of all DTC cases. To extend the lifespan of elderly PTC patients, it is critical to assess the risk factors for DSS and to predict prognosis. As previous studies primarily focused on adults in general, elderly patients (>=65 years old) with thyroid cancer were seldom studied. To address this, our study compared the clinical-pathologic features and DSS of DTC patients from 1988 to 2008 from the SEER database.

Patients with FTC were slightly older than patients with PTC. Certain demographic factors, such as gender and race, did not affect the DSS of all groups. The influence was histology-dependent. In addition, it may be partly attributed to the uneven demographic distribution, with females and Caucasians being the majority. Some studies attributed the higher incidence of thyroid cancer in women to an estrogen receptor that participates in cellular processes which enhanced tumorigenic properties of thyroid cells (15). Our study included primarily postmenopausal women, whose estrogen levels are low. Although it is possible that some of the women had undergone hormone replacement therapy, such a possibility does not detract from the need for additional studies that accounts for the sex bias observed herein.

With regards to the type of metastasis, certain studies have found that patients with PTC tend to primarily have lymph nodes metastasis, while patients with FTC tend to primarily have distant metastasis (9). A potential partial explanation for such a difference lies in the pathological feature of tumor—PTC mainly metastasizes through lymph nodes, whereas FTC spreads through hematogenous invasion (10, 16). Consistent with these findings, our study has found a higher prevalence for lymph nodes metastasis in C-PTC (22.2%) compared to FTC (6.7%), while that of FV-PTC (12.3%) lies between. Furthermore, the prevalence of distant metastasis in C-PTC and FV-PTC (3.6, 3.7%) was significantly less than in FTC (14.1%). As for the prevalence of lymph nodes metastasis, a reasonable comparison between the three DTC subtypes could not be made, since the status of lymph nodes metastasis for 19.9% of the cases in the FTC group was unknown. Moreover, as shown in Table 1, pathological examination of regional lymph nodes was quite low (15.3–36.8%). One of the possible explanations was that the status of lymph nodes metastasis was derived from ultrasound or CT scan, which were not documented within the SEER database.

As expected, survival analysis suggested that patients with FTC had the worst prognosis among the three DTC subtypes. Surprisingly, patients with FV-PTC had the highest disease-specific and overall-cause survival rate, as indicated by the Kaplan-Meier analysis. Such a finding is inconsistent with the tumor features of FV-PTC, given that previous studies had shown that FV-PTC had intermediate features, between those of C-PTC and those of FTC (9, 17, 18). Although some studies had suggested that FV-PTC could be more aggressive than C-PTC (19, 20), most studies concluded that the two subtypes have similar prognoses. The studies also found that, although the tumor features of FV-PTC are more aggressive than that of C-PTC, the two DTC subtypes had similarly good prognoses (9, 17, 18, 21). Studies that found FV-PTC to be more aggressive than C-PTC were usually observational studies with a small sample size; while the opposite view was supported by a large database. Consistent with latter studies, our study indicates that FTC lies between C-PTC and FTC in terms of its variety of clinical-pathological features, and has a longer survival compared to the other two subtypes.

That the clinical-pathological characteristics and the prognosis were inconsistent constitutes a paradox that can be potentially attributed to the histological pattern. More specifically, there are two main subtypes of FV-PTC: infiltrative FV-PTC and encapsulated FV-PTC, the latter of which is thought to be highly indolent. A number of studies from the past decade have found that patients with encapsulated FV-PTC had an excellent prognosis and were genetically distinct from patients with infiltrative tumors (22–24). In the most representative retrospective cohort study by Nikiforov et al., 109 patients diagnosed with non-invasive encapsulated FV-PTC had a very low risk of adverse outcome in a long follow-up, and most of the patients received only lobectomy (22). Based on the results of these studies, in 2016, what was formerly known as encapsulated FV-PTC was changed to NIFTP (non-invasive follicular thyroid neoplasms with papillary-like nuclear features) (22, 25). Most encapsulated FV-PTCs are still treated as conventional thyroid cancer. This abandonment of “cancer” in the formal designation of the disease was meant to deal with the problem of over-diagnosis and overtreatment of indolent thyroid cancers. Several studies reported that NIFTP occupied 20–25% of all tumors that were previously classified as thyroid malignancies (26, 27). Therefore, since it is likely that many FV-PTC cases were actually NIFTP, which had different tumor characteristics compared to infiltrative FV-PTC, it is not surprising that FV-PTC in this study showed the longest survival. On the other hand, the survival analysis suggested clinicians should consider histology when treating DTC, and be careful not overuse aggressive therapy, especially on elderly patients with comorbidity.

Using the Cox proportional hazards analysis, we assessed the association between specific risk factors and prognosis of DTC. Common risk factors among all three DTC subtypes included lymph nodes metastasis, extra-thyroidal extension, and distant metastasis, consistent with the conclusion drawn from most studies (8).

Although the traditional TNM staging system sets the age threshold for different stages as 45, more and more experts question the rationality of using the age 45 for risk stratification. In using age as both a categorical and a continuous variable risk predictor, some studies have found that advanced age considerably decreases the DSS of DTC, and that there was no age cut-off for significant risk stratification (28, 29). Both Jonklaas et al. (30) and Banerjee et al. (31) also emphasized the importance of age at diagnosis but questioned the rationality of a cut-off age. Recent international retrospective studies suggested an increase of the cut-off age from 45 to 55 (29, 32–34). Using only patients aged 65 or above, our study revealed that the risk of DSM would increase by a factor of 1.084–1.096 per year for DTC. For the elderly population, there was no cut-off point of age in decreasing survival. Although the newest edition of the AJCC stage system (8th edition) has increased the cut-off age to 55 years-old (32), whether the cut-off age of 55 is appropriate remains unknown. Perhaps, using age as a continuous variable without cut-off point may be more appropriate for predicting the outcome of DTC patients.

In terms of gender and race, previous studies have shown that males and/or blacks are high risk groups for DTC, and that marriage was a protective against DTC (5, 35). In our study, being male, being black, and being single were the only risk factors for DTC. Based on subgroup analysis, males have a higher risk of DSM from PTC but not from FTC. We determined that histology type has an impact on DSS. In particular, patients with FTC are more likely to be at high risk of baseline tumor features (Table 1), and presented with large tumor size, extra-thyroidal extension, and distant metastasis. FTC with aggressive tumor features and compromised survival was an important risk factor for DSM of DTC (HR 1.3, *p* = 0.004).

Lymph node metastasis, extra-thyroidal extension, and distant metastasis were all strong risk factors to the outcome of DTC patients. However, the precise role of lymph node metastasis in DTC remains controversial. An increasing number of studies have shown a significant decline of DSS in DTC in general (10). Both retrospective cohort analysis and large population-based database studies have reached inconsistent conclusions concerning the impact of lymph nodes involvement (36, 37). Our study has found that lymph nodes involvement was associated with a decrease in DSS for all three types of DTC.

As our study focused exclusively on patients who underwent surgery for DTC, we compared the effectiveness of thyroidectomy and lobectomy. Some previous analyses on DTC showed that there was no difference with respect to surgical method (6, 16). Consistent with these studies, we found that neither Cox regression analysis of DTC nor subgroup analysis showed any advantage of total thyroidectomy over lobectomy in terms of DSS. In addition, some studies using the SEER registry also demonstrated a lack of survival benefit from radiation therapy in patients with DTC (17, 38). Consistent with such findings, we have found that radiation therapy did not translate to a significant positive outcome in all three DTC subgroups (*p* > 0.05). Previously, there was no standardized RAI treatment. As such, we were not able to determine the effectiveness of RAI on ameliorating DTC. The 2015 American Thyroid Association Management Guidelines for Adult Patients with Differentiated Thyroid Cancer recommends lobectomy as the initial treatment option for DTC, and does not typically recommend RAI among low-risk patients (1). Therefore, clinicians should think twice before recommending aggressive treatment such as total thyroid or RAI in low risk patients. As the elderly population is often accompanied with other diseases such as diabetes and cardiovascular diseases, they are likely to bear additional risk, such as surgery complications and adverse reactions from RAI. Such information is not documented within the SEER database and therefore our assessment for the effectiveness of the therapeutic options remains incomplete. Hauch et al. (39) compared the effect of surgical method and found that total thyroidectomy corresponded to a higher risk of complications, even if the surgery was performed by high-volume surgeons. It is important for clinicians to assess risk stratification for elderly patients with DTC and avoid over-treatment.

The SEER database contains information on both overall-cause and disease-specific mortality, but not cancer recurrence. Therefore, the risk factors estimated by disease-specific survival may not be accurate for thyroid cancer recurrence. Although the SEER registries are representative of the cancer registries in the United States, it contains no information on the patients' comorbidities, clinical status, molecular markers, and drug therapy. Despite these limitations, however, large sample analysis using data derived from the SEER registries can help unveil the clinic-pathologic characteristics of tumors, thereby potentially leading to better options for prevention and treatment. With respect to elderly DTC patients, certain risk factors and the effectiveness of treatment remain controversial, and thus require more information and cases for further research.

In conclusion, patients with FTC, in comparison to those with PTC, tend to have a more advanced tumor stage as well as higher risk of specific clinical and pathological characteristics. Patients with FV-PTC require more specific histology catalog and risk assessment, both of which suggest a conservative therapy. Male, extra-thyroidal extension, lymph nodes metastases, and distant metastasis were all risk factors that predict a poor outcome in elderly DTC patients. Total thyroidectomy and RAI treatment appeared to confer no advantage in terms of lengthening disease-specific survival in elderly patients with DTC. We realized that the conclusions from SEER databases should not be overstated. Based on the present study, we recommend that risk stratification should be paid attention to and treatment should be individualized for elderly patients.

## Data Availability Statement

All data were obtained from the National Cancer Institute's Surveillance, Epidemiology, and End Results (SEER) Program database.

## Author Contributions

LY, JH, HZ, and XY: conceptualization. HH, HZ, and XY: methodology. LY, SL, HZ, and XY: writing. XY: supervision. HZ and XY: funding acquisition.

### Conflict of Interest

The authors declare that the research was conducted in the absence of any commercial or financial relationships that could be construed as a potential conflict of interest.
